# Scalable hybrid chemical manufacture to photothermal therapy: PEG-capped phototransducers

**DOI:** 10.1038/srep31351

**Published:** 2016-08-10

**Authors:** Jeong Hoon Byeon

**Affiliations:** 1 School of Mechanical Engineering, Yeungnam University, Gyeongsan 38541, Republic of Korea

## Abstract

Ag-TiO_2_@polyethylene glycol (PEG) nanoparticles were continuously obtained in a single-pass configuration by appropriately reacting freshly flame-synthesized TiO_2_ with Ag formed in an ultrasonic aqueous medium containing PEG. When the proposed synthesis was kept constant, the production rate for Ag-TiO_2_@PEG nanoparticles reached approximately 3 g/h while only using a combination of a lab-scale inverse-diffusion flame (16 mm head diameter) and an ultrasonic Ag(I) cell (50 mL). The synthesized nanoparticles were employed as inducers for *in vitro* photoinduced therapy to kill cancer cells at different light wavelengths. Measurements of the nanoparticle cytotoxicity revealed that PEG incorporation with the Ag-TiO_2_ particles significantly decreased the cytotoxicity (cell viability of more than ~91% at 200 μg mL^−1^ particle concentration) of Ag, and this was comparable with that of TiO_2_ particles (cell viability of more than ~90%). When 632 nm and 808 nm light was applied to the nanoparticles in the HeLa cells, the viability of the cells was significantly affected [decreased to ~4% (632 nm) and ~26% (808 nm) at 200 μg mL^−1^, 5 min irradiation time] by surface plasmon resonance heating and photothermal therapy.

Strategic incorporation between isomers may help enhance composite nanoplatforms, which can provide multi-functionalities in specific technological areas that are difficult to achieve with one-component nanoparticles. Therefore, it is important to develop improved metallic nanoparticles containing composite materials. In particular, there is an increasing interest in developing biocompatible functional materials for metallic nanoparticle technology, with an aim to combine the relevant properties of metals with the peculiar performance of biomaterials^1^. However, currently available metallic nanoparticles usually have poor compatibility with organic components, and it is therefore rather difficult to homogeneously incorporate them into an organic matrix. This limits the extension of their use in biomedical applications.

Numerous approaches have been proposed for fabricating multifunctional biocompatible nanomaterials that consist of inorganic-organic nanocomponents for application in diagnostic and therapeutic fields. These nanomaterials usually contain metallic nanoparticles as the core components with biofunctional polymers that endow the nanomaterial with unique physicochemical properties for diagnostic and therapeutic purposes^2^,^3^,^4^. Many formulations based on wet chemistry introduced as suspension of solid particles have been proposed for biomedical applications, and these may only be workable with desired performance in a short period of time^5^. Moreover, organic or polymeric components incorporated with metallic nanoparticles are normally unstable owing to gradual degradation by hydrolysis; biofunctional nanomaterials in a suspension or colloidal form would therefore not be recommended. As a result, there is a need for a paradigm shift in the strategy for preparing stable organic-inorganic hybrid nanomaterials with simpler and more versatile processing for efficient use in biomedical applications^6^. Gas-phase processing is an alternative method for preparing such nanomaterials with fewer preparation steps, and it could allow long-term storage of the prepared nanomaterials in a powder form^7^. Employing gas-phase processing further enhances the process continuity in production, and this implies that only simple mechanical collection of materials is required and large amounts of waste are not generated. Moreover, employing wet chemistry approaches has been recently shown to achieve better selectivity and safety in hazardous chemical processes and to exhibit many advantages over conventional batch chemical processes^3^.

Flame synthesis, which is one of the most promising gas-phase routes, has been utilized for preparing a wide range of mono- and multicomponent functional nanoparticles with low cost and high yield in commercial quantities^1^, and it could be scaled up to a production rate of several hundred grams per hour without significant change in material property^8^. However, conventional flame synthesis of functional nanoparticles is commonly performed in high temperature conditions (over 1,700 °C). Since temperatures over 300 °C can decompose most organic materials (i.e. biofunctional soft materials), the use of conventional flame synthesis would be only feasible to synthesize hard or inorganic nanoparticles^9^. Thus, for producing inorganic parts for hybrid nanomaterials is a single-pass configuration, flame pyrolysis would not be suitable without the need for post-treatment or post-functionalization steps; hence, this technique is still limited with respect to its use in a wide range of biomedical applications^10^. Nevertheless, more recently, flame-synthesized nanoparticles have often been employed as active materials for biomedical applications via successive incorporation with biocompatible overlayers in a continuous manner^1^,^6^,^11^. However, since this technique works only for inorganic biomaterials, its application is still limited for generalized use. There have been several studies on biomedical applications using metallic core-polymer shell nanocomposites that exhibit high absorption at wavelengths longer than that of visible light. Even though there are previous reports regarding organic matter/polymer incorporation with flame-synthesized nanoparticles^12^,^13^, it is still desirable to conceive of simple, continuous, and efficient methodologies for biomedical applications of flame-synthesized nanoparticles.

This work introduces the potential use of Ag-TiO_2_@polyethylene glycol (PEG) nanoparticles as photoinducers for killing cancer cells via flame-based gas-liquid-gas single-pass processing. The flame-synthesized TiO_2_ nanoparticles were employed as a biocompatible support^14^ to produce Ag-TiO_2_ nanobunches in order to enhance photoinduced cancer cell killing by exploiting localized surface plasmon resonance including photocatalytic activity^15^. To functionalize the flame-synthesized TiO_2_ particles with organic components in a continuous manner, an ultrasound Ag(I)-PEG reaction cell is employed both for efficient hydrosolization of the TiO_2_ particles and for subsequent incorporation with Ag and PEG on the TiO_2_ domains (Fig. 1). PEG is employed as overlayers for the Ag-TiO_2_ nanoparticles since it is known to be nontoxic to mammalian cells (i.e. biocompatible) and non-antigenic^16^, which may also help it bond with metallic ions^17^.

## Results and Discussion

To understand the formation of the Ag-TiO_2_@PEG nanocomposites through the proposed hybrid processing, the samples electrostatically deposited as TiO_2_, Ag@PEG, and Ag-TiO_2_@PEG [(using a commercial particle collector (NPC-10, HCT, Korea)] directly on carbon-coated copper grids in the gas-phase were analyzed using a transmission electron microscope (TEM, JEM-3010, JEOL, Japan). The gas-phase sampling flow rate was of 1.0 L min^−1^ in the collector. As shown in Fig. 2a, the primary TiO_2_ particles (~28 nm lateral dimension) were agglomerated, implying that the primary particles collided with each other after they formed near the flame. As seen in the inset of Fig. 2a, a lattice spacing of approximately 0.35 nm was most frequently observed, which could be matched to the (101) plane of anatase TiO_2_^18^. In Fig. 2b (Ag@PEG sample), a darker contrast of spherical particles exhibits the Miller plane (111) owing to a 0.24 nm face-centered cubic (fcc) Ag lattice (inset), whereas lighter-contrast overlayers on the spherical particles are attributable to the PEG. Thus, it was clearly observed that ultrasonically formed Ag (from Ag^+^ to Ag^0^) particles (8.8 ± 1.7 nm lateral dimension) could be successively incorporated with PEG via successive reaction and electrospray processes. In the case of Ag-TiO_2_@PEG, as shown in Fig. 2c, the different particles (different size and gradation) are combined as a single agglomerate, and it was clearly observed that darker dots (because of Ag’s higher density than TiO_2_) were randomly located on the TiO_2_ domains, which confirmed that the Ag-TiO_2_ hybrid structures were successfully assembled. The lattice spacing of the darker dots was ~0.24 nm corresponding to the Miller plane (111) of Ag, while that of the lighter-contrast dots was ~0.35 nm corresponding to the (101) plane of TiO_2_, implying coupling between distinct phase domains. The corresponding fast Fourier transform images are also attributable to the fcc Ag and anatase TiO_2_, respectively (Fig. 2d). As further evidence, the X-ray diffraction (XRD, RINT-2100, Rigaku, Japan) analysis of the TiO_2_ and Ag-TiO_2_ particles revealed that Ag particles formed in the aqueous solution collided with incoming TiO_2_ particles (Supplementary Fig. S1a). This implies that the process enables incorporation of Ag particles at several sites on the continuously incoming TiO_2_ particles. Furthermore, the Ag agglomerates (shown in Fig. 2b) were redistributed on the TiO_2_ domains due to deagglomeration (Eq. S1, Supplementary Information, by setting the mechanical force from a pressure drop on an agglomerate of certain size owing to the remarkable changes in pressure between the both sides of a nozzle for the electrospray). When the agglomerated Ag@PEG passes through the nozzle, it undergoes significant changes in mechanical conditions such as pressure and velocity across the nozzle which induce a structural change of the Ag@PEG before entering the nozzle. Figure 2c also displays the incorporation of PEG on Ag-TiO_2_ particles, which may have originated both from the Ag-PEG binding during Ag formation (Eq S2, Supplementary Information) in the ultrasound reaction cell and from adsorptive incorporation during electrospray. To confirm the latter, adsorption isotherms (Supplementary Fig. S2) were measured using the Micromeritics ASAP 2010 apparatus. A difference in the pore size distribution (inset) also supports the PEG incorporation, which shows a significant decrease in the pore volume at meso- and macro-porous regions after the PEG incorporation, implying that this adsorptive property also might enhance the PEG incorporation on the Ag-TiO_2_ particles^19^. Figure 2d shows the energy dispersive X-ray (EDX, JED-2300, JEOL, Japan) spectrum for the Ag-TiO_2_@PEG sample. The low- and high-magnification scanning electron microscopy (SEM, JSM-6500F, JEOL, Japan) images and the EDX spectrum peaks correspond to Ag, Ti and O, and arrows in the high-magnification image indicate the positions of Ag, Ti, and O in the sample. The weight fraction of Ag in the Ag-TiO_2_ was ~40.1% in the present work, and this further confirmed the Ag incorporation on TiO_2_ domains. When the system operation is kept constant, the production rate for Ag-TiO_2_@PEG particles reached approximately 3 g/h, and scale-up of this system may achieve higher production rates (from kilograms to tons per day).

The formation of Ag-TiO_2_ particles was also verified by means of UV-vis absorption spectroscopy (330, Perkin-Elmer, US, Supplementary Fig. S3a). This is also attributed to the dispersity of the Ag particles on the TiO_2_ domains by the ultrasonic Ag(I) reduction and this induces an enhancement in the Ag particle dispersity owing to increased interaction between the Ag and TiO_2_ particles, resulting in a broadening of the absorption. The inset of Supplementary Fig. S3a shows the X-ray photoelectron spectroscopy (XPS, AXIS HIS, Kratos Analytical, Japan) spectrum of the Ag-TiO_2_ nanoparticles. The characteristic peaks at 458.1 eV and 463.7 eV shown in Supplementary Fig. S1b are attributable to Ti 2*p*_3/2_ and Ti 2*p*_1/2_, respectively. There are two more characteristic peaks at 367.5 eV and 373.5 eV in Supplementary Fig. S1b, which are attributable to Ag 3*d*_5/2_ and Ag 3*d*_3/2_, respectively, proving the existence of a metallic Ag. Supplementary Fig. S3b displays Fourier transform infrared spectra (FTIR, IFS 66/S, Bruker Optics, Germany) of the TiO_2_, Ag@PEG, and Ag-TiO_2_@PEG samples. The bands around 1,270 and 1,005 cm^−1^ for the Ag@PEG and Ag-TiO_2_@PEG samples may have arisen from C-O, C-O-C stretches, and C-O-H bendings vibrations of the Ag particles in the PEG matrix^20^.

Flame-synthesized TiO_2_ particles were successively injected into an aqueous Ag(I)-PEG solution in the presence of ultrasound to achieve bubble implosion for efficient collection. To verify the quantitative collection of TiO_2_ particles, we measured the size distributions both in the absence and the presence of ultrasound application. Most incoming TiO_2_ particles were trapped (~98.5% efficiency) in the aqueous solution during ultrasound irradiation. This may be due to bubble implosion before they reach the surface of the solution, resulting in hydrosolization of nearly all TiO_2_ particles. The particle size distribution was analyzed using a scanning mobility particle sizer (SMPS, 3936, TSI, USA) to verify the number concentration, mean diameter, and standard deviation of the electrosprayed Ag-TiO_2_@PEG particles. The measured concentration, diameter, and deviation were 7.09 × 10^6^ particles cm^−3^, 125.0 nm, and 1.95, respectively, as shown in Fig. 3. The same data for TiO_2_ particles are 3.05 × 10^6^ particles cm^−3^, 61.1 nm, and 1.84, respectively, and for the atomized Ag@PEG particles are 5.32 × 10^6^ particles cm^−3^, 115.8 nm, and 1.97, respectively. The data for the Ag-TiO_2_@PEG particles were closer to that for the Ag@PEG particles compared to that of the TiO_2_ particles. There was no additional peak and only an apparent increase in concentration, not in size, suggesting that the TiO_2_ particles were well merged with the Ag particles and thus forming Ag-TiO_2_@PEG nanoparticles. In addition, the mass fractions of Ag, TiO_2_, and PEG in the Ag-TiO_2_@PEG nanoparticle were measured using a piezobalance particle monitor (3522, Kanomax, Japan) to be 0.38, 0.46, and 0.16, respectively.

We tested the cytotoxicity of the TiO_2_, Ag-TiO_2_, and Ag-TiO_2_@PEG nanoparticles. In the case of Ag-TiO_2_, a part of the dispersion was placed on HeLa cells to compare the cytotoxicity among the samples. The MTT [3-(4,5-dimethylthiazol-2-yl)-diphenyltetrazolium bromide] assay was employed to evaluate cell viability by using incubation cells with samples for 24 h (Fig. 4a). Measurements of cell viability showed that the TiO_2_ nanoparticles had a cell viability of ~90% at 200 μg mL^−1^, which indicates biocompatibility^21^, whereas the measured viabilities of the Ag and Ag-TiO_2_ nanoparticles were significantly lower than that for the TiO_2_ nanoparticles. The higher cytotoxicities for the Ag and Ag-TiO_2_ particles may be due to the release of Ag ions in water from the Ag particles (8.8 nm), and the released fraction of Ag ions reached approximately 38.4 wt% (using inductively coupled plasma optical emission spectrometry, Optima 8300, PerkinElmer, USA), which is consistent with a previous report (4~9 nm, Ag particle size)^22^. Analogous data for Ag-TiO_2_@PEG nanoparticles has the highest cell viability, i.e. ~92% at 200 μg mL^−1^. Although Ag-TiO_2_ nanoparticles released the metallic component to clearly contribute toward cytotoxicity, the released fraction of the PEG incorporated particles were not affected significantly because of the biocompatible organic overlayers, as shown in Fig. 2c. This tendency is consistent with a previous report, which described that protective PEG overlayers could decrease the cytotoxicity of nanoparticles to mammalian cells^23^,^24^. This suggests that the Ag-TiO_2_@PEG nanoparticles from the successive Ag-PEG incorporation of flame-synthesized TiO_2_ particles warrant further investigation for their usage as photoinducers for HeLa cell killing. The zeta potential of Ag-TiO_2_@PEG nanoparticles was +2.2 mV at pH 7.4, and this value was relatively more positive than that for the PEG-capped Ag particles prepared by wet chemistry^25^, hence this may provide higher affinity with negatively charged biological membranes. According to a common protocol for testing the photothermal activity of Ag-TiO_2_@PEG nanoparticles, we first measured the temperatures of the media in the presence of light irradiation for a duration of 5 min. The nanoparticle solution embedded in agar was exposed to laser of wavelength 632 nm or 808 nm (2.15 W cm^−2^). These wavelengths were chosen since they are within the absorption range of the Ag-TiO_2_ nanoparticles (Supplementary Fig. S3a). The absorption spectra of Ag-TiO_2_ particles showed a significant shift towards the longer wavelength (480~650 nm) compared with TiO_2_ particles, which is comparable with results from a previous report^26^. The broadened and shifted spectra may be due to the surface plasmons of Ag particles that depend on the morphology (i.e., nanobunches) and surrounding environment. The red shift in the spectra may have resulted from the interaction between Ag and TiO_2_ particles, where the Ag particles were deposited on the TiO_2_ surface^27^. The temperature, which was measured using an IR thermometer with a thermal camera (Supplementary Fig. S4), increased with an increase in laser irradiation time, and the local temperature reached 51.5 °C. Hence, the maximum values (23.1 °C at 632 nm and 16.3 °C at 808 nm) of *∆T* (Eq S3, Supplementary Information) could be realized at 200 μg mL^−1^ of mass concentration, while the analogous value for the 365 nm wavelength was 0.2 °C. From the temperature measurements, it can be concluded that the Ag-TiO_2_ nanoparticles absorbed the irradiated laser light, and the light was successively converted into thermal energy. Based on this photothermal conversion of the Ag-TiO_2_ particles upon 632 nm or 808 nm excitation, *in vitro* photothermal therapy using Ag-TiO_2_@PEG nanoparticles was investigated. To measure the photothermal activity of the nanoparticles for cell killing (Fig. 4b), adenosine triphosphate (ATP) assay was employed. The thermal energy obstructed the ATP production of the cells owing to the heat when the laser irradiated the culture medium. According to previous reports^28^,^29^,^30^, the positive effect of temperature on photoinduced reactions in the presence of plasmonic and nonplasmonic noble metal nanoparticles is interpreted in terms of the redistribution of the metal electron into higher energy levels and the increase in vibrationally excited states with increasing temperature. Thus, the cell viability significantly decreased from thermo/chemical reactions with the embedded nanoparticles. Live or dead cells were differentiated by calcein AM (live cells, green fluorescence, Life Technologies, USA) and propidium iodide (dead cells, red fluorescence, Life Technologies, USA) after laser irradiation (inset of Fig. 4b). In the control group (Ag-TiO_2_@PEG nanoparticles only), nearly all cells displayed green fluorescence, so the particles themselves have no significant cytotoxic effects. When the nanoparticles are exposed to laser with different wavelengths, the particles show a photothermal effect on HeLa cells; hence, only cells within the laser spot were killed, showing homogeneous red fluorescence. The cells outside the region of the spot were mostly kept alive, showing green fluorescence. In particular, when the concentration is 90 μg mL^−1^, the viability of the cells incubated with Ag-TiO_2_@PEG nanoparticles decreased significantly with irradiation (2.15 W cm^−2^), and more than 80% of the cells were killed within 5 min of irradiation (632 nm). This performance is comparable with that in a previous report^31^ where modified TiO_2_ nanoparticles were employed with 5 min of irradiation (2.00 W cm^−2^). Even though there is a difference in the maximum temperature between the current and previous results for 5 min of laser irradiation, both results reveal temperatures over 50 °C that can easily kill cancer cells^32^. This implies that the proposed approach has feasibility that may be investigated further. In addition, the analogous result for the 365-nm wavelength did not show significant enhancement (more than ~80% in cell viability even at 200 μg mL^−1^) in cell killing, implying that the produced heat was rather critical for the cytotoxic effect. The weaker performance at the UV case may be due to significant UV absorption (~70% for <400 nm wavelength) by a silicon-coated polyethylene terephthalate (PET, 125-μm thickness, Loparex, USA) film^33^ that was employed to simulate the laser irradiation on skin^34^,^35^.

Furthermore, another scenario for parenteral applications was considered, where the Ag-TiO_2_@PEG nanoparticles are administered to tissues that attract activated macrophages. In order to suppress inflammatory responses, nanoparticle interaction with biological system has recently been studied for biofunctional nanoparticulate systems^36^. The results (Fig. 5) show that the Ag-TiO_2_@PEG nanoparticles could more significantly suppress the macrophage inflammatory protein (MIP) production from lipopolysaccharide (LPS)-challenged macrophages than those from polyethyleneimine (PEI) or poly-l-lysine (PLL) [as well as phosphate buffered saline (PBS)] incorporated Ag-TiO_2_ nanoparticles (insets of Fig. 5). The smaller MIP productions of the Ag-TiO_2_@PEG nanoparticles than that from the PEI or PLL incorporated particles indicate that the tendency may be related to the amine content (i.e., no amine groups in PEG).

A continuous aqueous Ag-PEG capping of flame-synthesized TiO_2_ nanoparticles via gas-liquid hybrid processing was employed for the first time for scalable production of inorganic-organic nanoplatforms. The fabricated nanoplatforms were directly employed to evaluate their biocompatibility and photothermal activity for killing cancer cells. A further study to optimize the proposed method for realistic applications regarding modulation of optical property and cytotoxicity is now in preparation for publication elsewhere. This strategy could allow on-demand fabrication of fresh biomaterials for use in biomedical applications, and may also hold immense promise in biomaterial coatings. These results further establish continuous hybrid processing as an efficient, green, and scalable design and fabrication methodology, which is generalizable to a wide range of therapeutic and diagnostic agents for both biomedical and scientific purposes.

## Materials and Methods

**Figure 1:**
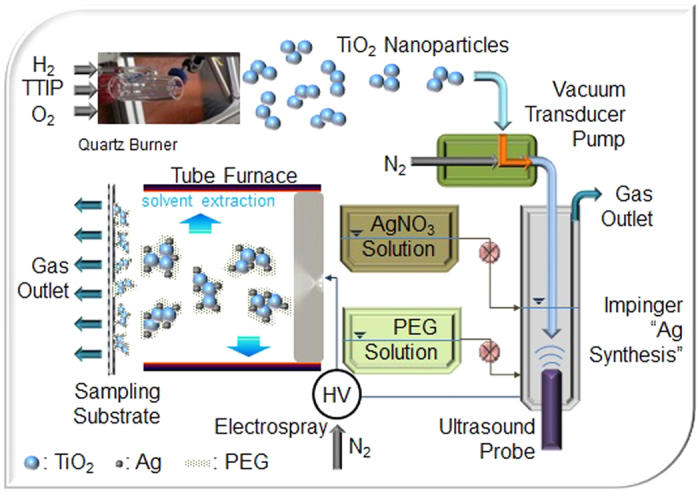
Schematic of gas-liquid hybrid route for continuous fabrication of Ag-TiO_2_@PEG nanoplaforms with a burner, Ag-PEG solution reactor, and electrohydrodynamic atomizer connected in series.

**Figure 2:**
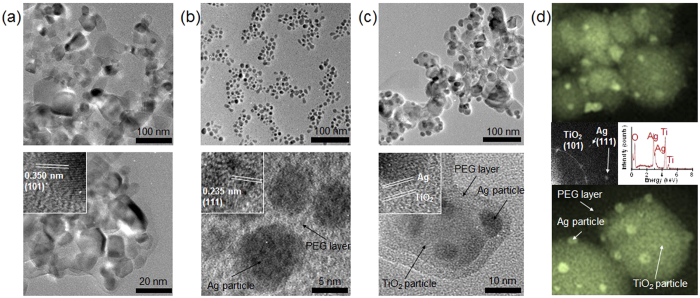
Low- and high-magnification electron microscope images to verify the morphologies of the synthesized particles: (**a**) flame-synthesized TiO_2_ nanoparticles; (**b**) ultrasound-assisted polyol synthesized-electrosprayed Ag@PEG nanoparticles; (**c**) ultrasound-assisted-electrosprayed Ag-TiO_2_@PEG nanoparticles; and (**d**) SEM images and corresponding EDX profile, and FFT image for Ag-TiO_2_@PEG nanoparticles.

**Figure 3:**
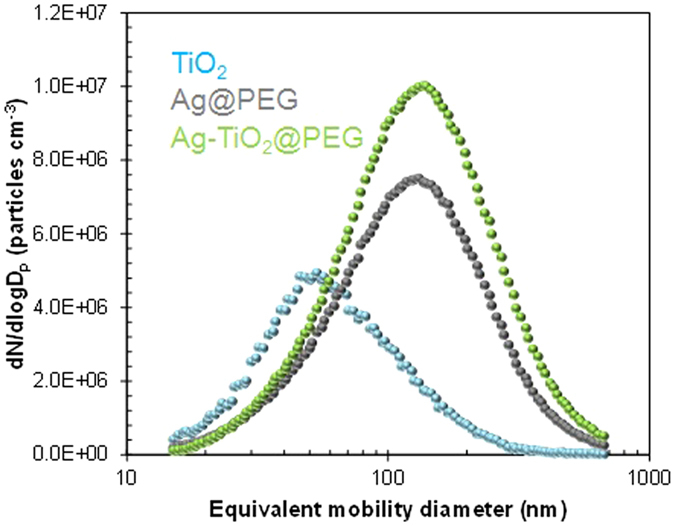
Particle size distributions of azerosol TiO_2_, Ag@PEG, and Ag-TiO_2_@PEG nanoparticles.

**Figure 4:**
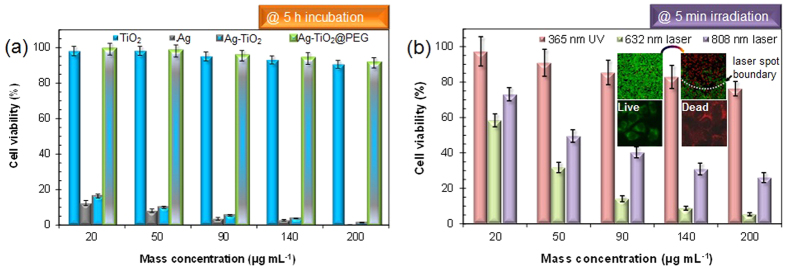
Biological analyses regarding photothermal activity of Ag-TiO_2_@PEG nanoparticles: (**a**) cytotoxicity of TiO_2_, Ag, Ag-TiO_2_ and Ag-TiO_2_@PEG nanoparticles; and (**b**) photothermal activity for killing HeLa cells upon irradiation with different wavelengths.

**Figure 5:**
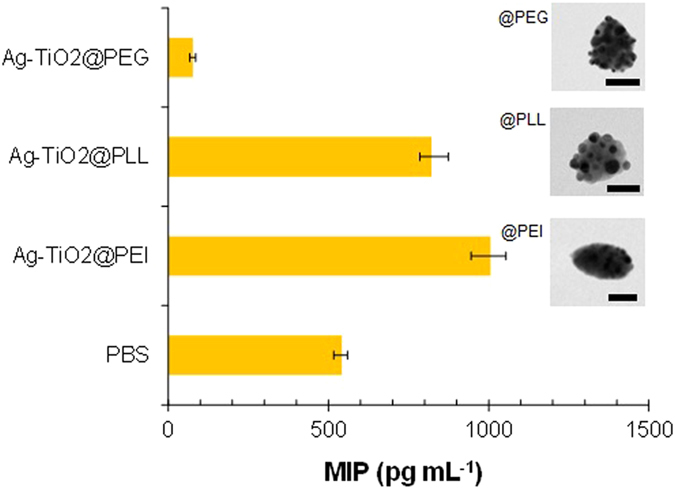
MIP production from LPS-challenged macrophages by adding PEI, PLL, and PEG incorporated (2 × 10^−6^ mol dm^−3^) Ag-TiO_2_ nanoparticles. Insets show representative TEM images (scale bar, 50 nm) of the polymer incorporated Janus particles.

Nanoparticles synthesized in the gas-phase can be suspended into a liquid for coupling to wet-chemistry routes, thus enabling development of other innovative methods for novel material synthesis^37^,^38^,^39^. When incorporating metal in a liquid state, hydrated electrons produced during mixing and heating could reduce metal ions to metal particles of zero valences. In the present work, the flame-synthesized TiO_2_ inorganic particles were injected into an Ag(I)-PEG containing aqueous solution in the presence of ultrasound for capping of the TiO_2_ particles with newly formed Ag-PEG (Fig. 1). The TiO_2_ particles were ultrasonically trapped in the solution when the ultrasound reached the bubbles containing TiO_2_ particles in the solution. When solutions containing AgNO_3_ and PEG were separately injected into the reactor containing TiO_2_ particles, the Ag ions would reduce and subsequently deposit on the trapped TiO_2_ particle by ultrasound-assisted reaction and mixing. They were then aerosolized via electrospray to fabricate nanoscale PEG incorporated Ag-TiO_2_ photoinducers, and finally employed to kill human epithelial carcinoma (HeLa, ECACC No. 93021013) cells upon light irradiation (with a 632 nm or 808 nm wavelength, VA-I-DC, Rhysics Phototronics, Korea).

Specifically, as shown in Fig. 1, a commercial co-flow quartz burner (141/18/60, Arnold Gruppe, Germany) equipped with a bubbler was employed to produce TiO_2_ nanoparticles. The H_2_ (fuel) intake is made through 4 capillaries, and the TiCl_4_ precursor (208566, Sigma-Aldrich, USA) was fed to the flame via bubbling air passing around the 4 capillaries after mixing with another air acting as oxidant and sheathing gas. The H_2_ flow rate was 1.2 L min^−1^, and the air flow rates for bubbling and oxidation were 0.3 and 6.0 L min^−1^, respectively. The flow rates were separately controlled with mass flow controller (Tylan, USA). The synthesized TiO_2_ particles passed through the tube furnace containing silica gel and activated carbon pellets to remove extracted solvent and were driven to a conical chimney, which directly drove the part of particles using a vacuum transducer pump into the ultrasonic the Ag(I) cell containing PEG to employ as domains for synthesizing Ag-TiO_2_ nanoparticles in Ag(I)-PEG solution. In the reaction cell, solutions 1 and 2 were injected with the aid of a peristaltic pump (323Du/MC4, Watson-Marlow Bredel Pump, USA) at constant rates of 0.42 and 2.08 mL min^−1^, respectively. 85 milligrams of AgNO_3_ (205052, Sigma-Aldrich, USA), used as a precursor of Ag, was dissolved in 5 mL of deionized water (Solution 1). 0.2 grams of PEG (81253, Sigma-Aldrich, USA), used both as a reducing agent and a stabilizing agent, dissolved in 25 mL of distilled water (Solution 2). An ultrasound probe (VCX 750, 20 kHz, Sonics & Materials Inc., USA) was immersed into the solution. The probe acted as an ultrasound irradiator (10 W mL^−1^ input power density) and the active part of the probe was the planar circular surface, of area 1.3 cm^2^, at the bottom of the probe. Upon the start of reactions, the pale yellow solution changed to light brown indicating the reduction of Ag(I).

The Ag-TiO_2_@PEG suspension was aerosolized via electrospray, by means of the equipment assembled in-house. In brief, the equipment consisted of another peristaltic pump (07522-30, Masterflex, USA), a stainless-steel nozzle (inner diameter 0.3 mm), a high-voltage power supply (10/40A, Trek, USA), and a stainless steel plate placed directly below the nozzle as the grounded count electrode at a distance of 40 mm between the capillary and the substrate. The prepared solution was electrosprayed (16 μL min^−1^) in a heated tube installed between a capillary and a polytetrafluoroethylene substrate (66042, Satorius, Germany) using the pump (3032, TSI, USA) to perform a deposition of Ag-TiO_2_@PEG nanoparticles on the substrate. The detached nanoparticles from the substrate were then employed to evaluate their cytotoxicity and photothermal activity upon light irradiation.

Synthesized Ag-TiO_2_@PEG nanoparticles were evenly dispersed in 2% agar (Invitrogen, USA) at concentrations of 20, 50, 90, 140, and 200 μg mL^−1^. The gels were formed in shallow, 35 mm diameter plastic petri dishes. For exposure, the gel phantom samples at room temperature were exposed to a photoirradiation system passing through a silicon-coated PET film (to simulate the laser irradiation on skin) with different wavelength ranges (365, 632, and 808 nm). The gel samples were positioned in the light and irradiated by the beam (2.15 W cm^−2^ irradiation intensity) for a fixed duration of 5 min. In order to evaluate the application to photothermal cell killing, ATP assay (BioVision, USA) was employed, which is based on a highly sensitive firefly reaction to determine the level of cellular ATP as a surrogate marker for the number of live cells. After a 24 h incubation with Ag-TiO_2_@PEG nanoparticles, the cells were washed three times with a Hank buffered salt solution (H9394, Sigma-Aldrich, USA), and 0.1 mL of CellTiter-Glo Luminescent (Promega, USA) assay reagent was added to each well, and the plate was then mixed using an orbital shaker for 2 min, followed by 10 min incubation to stabilize the luminescence signals. Luminescence was read using a luminometer (GloMax 20/20, Promega, USA).

Peritoneal macrophages (ATCC CRL-2457) were seeded in 24-well plates at a density of 10^5^ cells per well in 1 mL of medium. After overnight incubation, 0.1 mL of the Ag-TiO_2_@PEG nanoparticle solution was injected to each well to set the particle concentration in medium to 2 mg mL^−1^. For comparison purposes, 0.1 mL of polyethyleneimine (PEI, 765090, Sigma-Aldrich, USA), poly-l-lysine (PLL, P4707, Sigma-Aldrich, USA), or PEG was injected in lieu of the Ag-TiO_2_@PEG nanoparticle solutions. After 24 h incubation, the culture media were centrifuged at 2000 rpm for 10 min to separate supernatants. Macrophages were challenged by adding lipopolysaccharide (LPS) to the media in the final concentration of 1 μg mL^−1^ shortly before the comparisons. Enzyme-linked immunosorbent assay (ELISA) was performed to determine the MIP levels using MIP-2 ELISA kit (R&D Systems, USA). The supernatants collected from LPS-challenged macrophages were always diluted 10 times prior to the analysis. The differences were considered significant for *p* < 0.01.

All experiments were performed in triplicate, and the results were reported as average values and standard deviations.

## Additional Information

**How to cite this article**: Byeon, J. H. Scalable hybrid chemical manufacture to photothermal therapy: PEG-capped phototransducers. *Sci. Rep.* **6**, 31351; doi: 10.1038/srep31351 (2016).

## Supplementary Material

Supplementary Information
