# Supplementary material for: Scalable hybrid chemical manufacture to photothermal therapy: PEG-capped phototransducers
Source: Sci Rep. 2016 Aug 10;6:31351. doi: 10.1038/srep31351 (PMC4979092; doi:10.1038/srep31351)
Supplement: Supplementary Information [file srep31351-s1.pdf]

Scalable hybrid chemical manufacture to photothermal therapy: PEG-capped phototransducers

Jeong Hoon Byeon[*]

School of Mechanical Engineering, Yeungnam University, Gyeongsan 38541, Republic of Korea

Equation for Deagglomeration (D_{pr})

$$D_{pr} = \alpha \sqrt{\frac{D_{pa} H}{6\pi \Delta P \Theta^2}} \tag{S1}$$

where D_{pr} is the size of a restructured agglomerate, α is the proportionality constant, H is the Hamaker constant, ΔP is the pressure difference between the front and the rear of the nozzle, Θ is the parameter controlling the maximum cohesive strength between the constituting particles in an agglomerate.

Chemical Reaction for Ag Formation

Ag^{+} + PEG → $[Ag(PEG)]^{+}$

$2[Ag(PEG)]^{+}$ + $CH_2OH(CHOH)_4CHO$ → $2[Ag(PEG)]^{0}$ + $CH_2OH(CHOH)_4COOH$ (S2)

EQUATION FOR LIGHT-INDUCED HEATING (ΔT)

$$\Delta T\left(D_p\right) = \frac{V_p P_{abs}}{2\pi k_0 D_p} \tag{S3}$$

where D_p and V_p are the diameter and volume of the nanoparticles, respectively, k_0 is the thermal conductivity of the surrounding liquid, and P_{ab} is the local light-induced heating of the nanoparticles.

Fig. S1

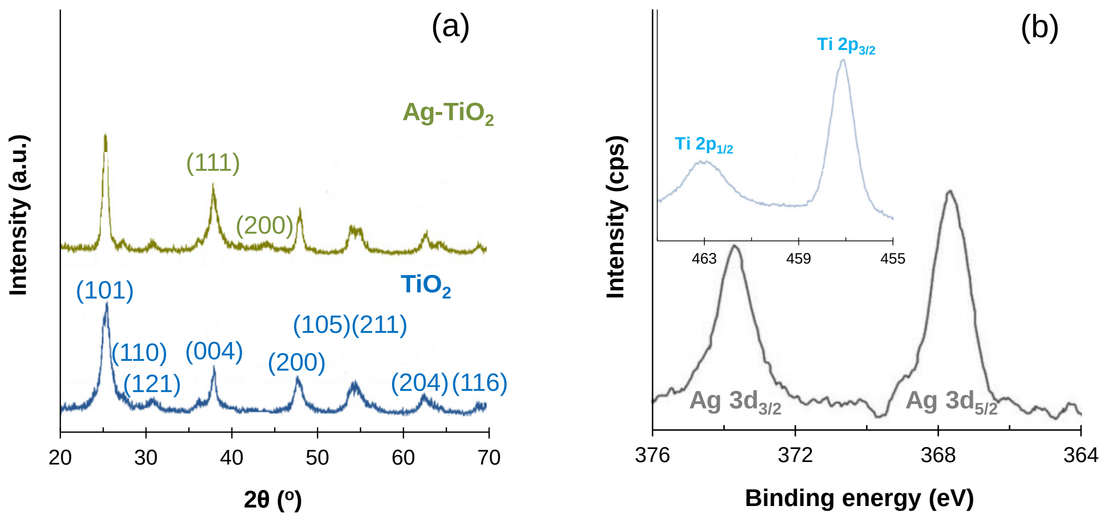

(a) XRD spectra of TiO_2 and Ag-TiO_2 nanoparticles. (b) XPS spectra of Ag-TiO_2 nanoparticles.

The XRD profile shows bands at 2θ = 25.6°, 38.0°, 47.9°, 54.2°, 62.9°, and 69.1°, which were attributable to anatase TiO_2 (101), (004), (200), (211), (204), and (116) crystallite, respectively (JCPDS 21-1272). As estimated from the Debye-Scherrer equation for the (101) peak, the crystalline size of TiO_2 particles for the anatase was ~22 nm, which was consistent with the TEM observation (**Fig. 2**a). Meanwhile, the profile for Ag-TiO_2 did not distinctly show the (111) peak of Ag particles in the Ag-TiO_2 particles, because many parts of the Ag particles were covered with the intense peak for the (004) peak of anatase TiO_2 which is located at 38.0° and is very close to the (111) peak (38.1°) of Ag (JCPDS 04-0783). Nevertheless, no significant changes in the peak angle in the diffraction were verified, implying that Ag was physically located on the TiO_2 particles via ultrasound-assisted assembly of Ag-TiO_2 nanoparticles.

Fig. S2

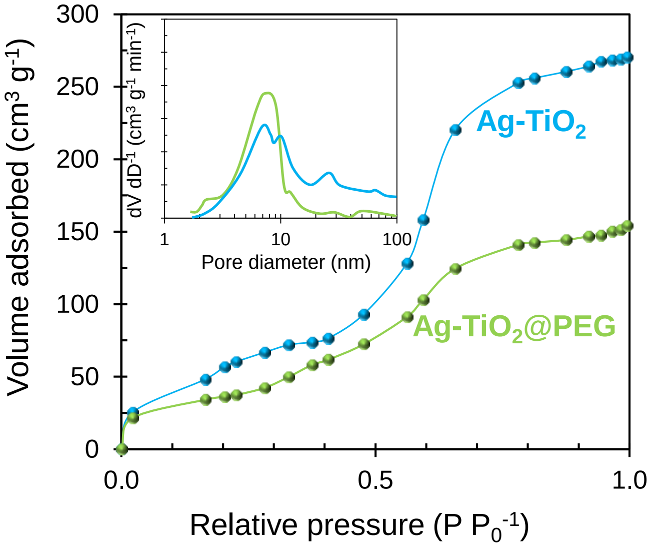

Adsorption isotherms of Ag-TiO_2 and Ag-TiO_2@PEG nanoparticles including their pore size distributions (inset).

The two distinct adsorptions at over 0.8 and under 0.8 of P P_0^{-1} may be attributed to the voids between the Ag-TiO_2 hybrid structures and the internal region of the particles, respectively. The pore reduction is also attributable to the fact that Ag particles were dispersed within the pore channels of the TiO_2 domains, leading to the blocking of the pore channels.

Fig. S3

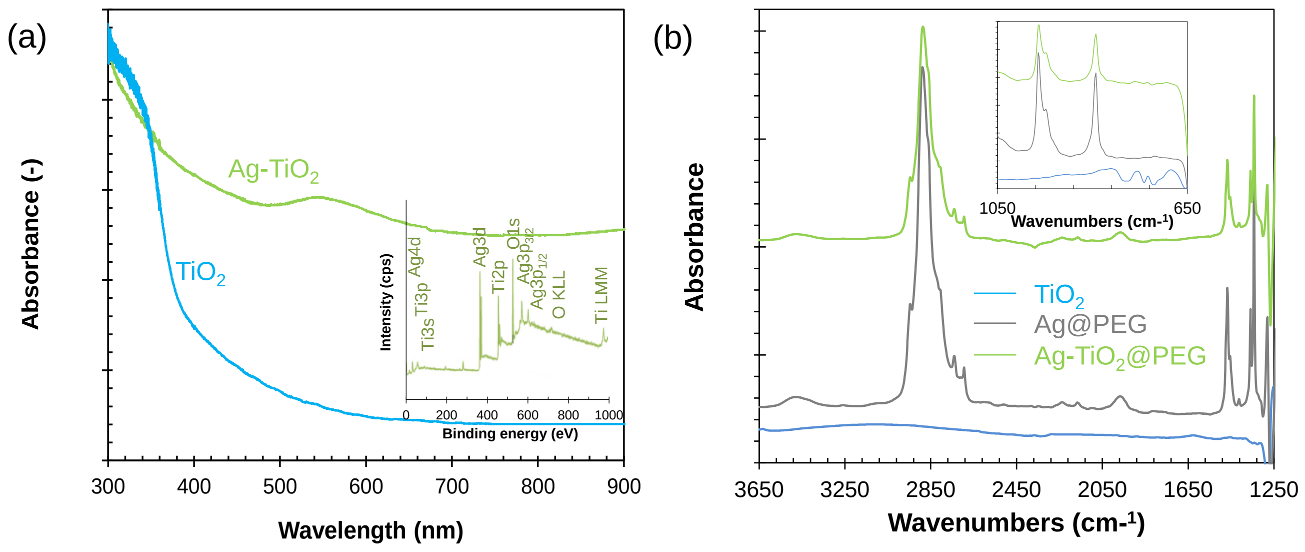

Structural analyses of synthesized nanoparticles: (a) UV-vis and XPS (inset) spectra of TiO_2 and Ag-TiO_2 nanoparticles; and (b) FTIR spectra of TiO_2, Ag@PEG, and Ag-TiO_2@PEG nanoparticles.

The spectra (**Fig. S3**a) of nanoparticles show that incorporation of Ag on TiO_2 produces a new broad band at ~570 nm, which would be attributed to the effect of localized surface plasmon resonance on the Ag components. The two depicted peaks for the Ag-TiO_2 case confirmed the existence of both the TiO_2 and Ag components, respectively. It can be seen that the plasmon resonance peak for Ag shifts to a longer wavelength, implying that the size and morphology of the Ag particles are changed.

The peaks (inset of **Fig. S3**a) in the profile can be attributed to Ti, O, and Ag elements corresponding to the Ag-TiO_2 particles. The profile also contains a peak (529.7 eV) attributed to the O 1*s* (ascribed to lattice O^{2-} of TiO_2), and this implies that the ultrasonic incorporation of Ag did not lead to any shift in the O 1*s* characteristic peak (cf. pure TiO_2 of 529.7 eV), disproving the existence of Ag oxides. However, the characteristic peaks somewhat shifted compared to the reference value of bulk Ag (368.2 eV), the $3d_{5/2}$ peak of Ag in the present work shifted downward in binding energy. This may be due to the ultrasonic interaction between the Ag and TiO_2 crystallites. When the Ag and TiO_2 collide with each

other, electrons were transferred from TiO_2 to the Ag particles owing to the different Fermi energy levels of the two components.

In addition, as shown in **Fig. S3**b, the aliphatic C-H stretching at around 1,415 and 1,345 cm^{-1} were due to C-H bending vibrations. The characteristic peak at around 1,950 cm^{-1} is related to the substituted allene groups, which also might be the products of PEG oxidation to reduce Ag(I), while the bands at around 800 cm^{-1} (inset of **Fig. S3**b) could be attributed to aromatic out-of-plane C-H bending. The peaks at around 2,900 cm^{-1} were attributed to the aliphatic C-H stretching, and those at around 1,440, 1,375, and 1,340 cm^{-1} may be due to C-H bending vibrations. The absorption band at arround 3,420 cm^{-1} was attributed to the O-H stretching band, and the intensity of the stretching band decreased when Ag-TiO_2 was loaded to the PEG, suggesting the chelation of Ag including TiO_2 with the hydroxyl groups of PEG. This also implies that both Ag(-TiO_2) particles were conjugated well with PEG with bindings between Ag(-TiO_2) and hydroxyl groups. The inset of **Fig. S3**b also shows the incorporation of TiO_2 domains, and the band intensities attributed to Ti-O and TiO_2 stretching observed at around 730 cm^{-1} are lower than those of the TiO_2 sample, proving that TiO_2 crystallites are located within the Ag-TiO_2@PEG.

Fig. S4

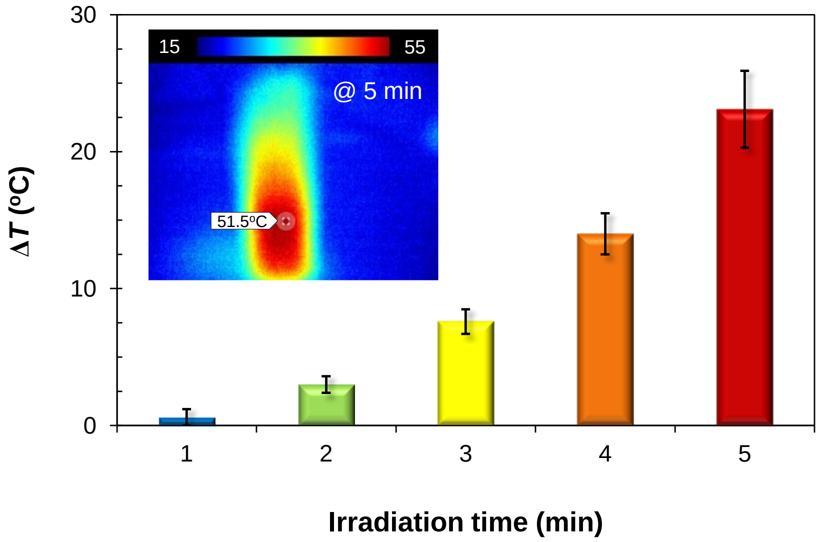

Temperature elevations (ΔT) upon laser (632 nm wavelength) irradiation for 1-5 min. The starting temperature was 28.4 ^{o}C.

Table S1 Details of the size distributions of flame-synthesized TiO_2, and electrosprayed Ag@PEG and Ag-TiO_2@PEG nanoparticles

Case	GMD (nm)	GSD (-)	TNC (× 10^6 particles cm^{-3})
TiO_2	61.1±2.2	1.84	3.05
Ag@PEG	115.8±3.9	1.97	5.32
Ag-TiO_2@PEG	125.0±4.0	1.95	7.09
